# Trends in mortality and associated factors among neonates hospitalized at Muhimbili national hospital, Tanzania: A three-year retrospective study

**DOI:** 10.1371/journal.pone.0310256

**Published:** 2024-11-08

**Authors:** Tiwonge Msonda, Robert Moshiro, Nahya Salim, Helga Naburi

**Affiliations:** 1 Department of Pediatrics and Child Health, Muhimbili University of Health and Allied Sciences, Dar es Salaam, Tanzania; 2 Department of Pediatrics and Child Health, Muhimbili National Hospital, Dar es Salaam, Tanzania; 3 Department of Pediatrics and Child Health, University of Cape Town, Cape Town, South Africa; University of Zimbabwe Faculty of Medicine: University of Zimbabwe College of Health Sciences, ZIMBABWE

## Abstract

**Background:**

Tanzania is amongst the countries with high neonatal mortality in Sub-Saharan Africa (SSA), and estimates vary widely among regions. Various interventions are being implemented at Muhimbili National Hospital (MNH), a tertiary and teaching facility, to contribute towards the reduction of neonatal mortality. This study aimed to detail the magnitude, trends and factors associated with neonatal mortality at MNH.

**Methods and findings:**

A hospital-based retrospective cohort study was conducted from January 2018 to December 2020. Records of all neonates admitted during the study period were extracted from neonatal registers and the electronic medical record system and recorded in a pretested data collection form. Data cleaning and analysis were done using SPSS version 23. Poisson regression was used to determine adjusted relative risk (aRR) with a 95% confidence interval (CI) to test association. A *p*-value of <0.05 was considered significant. Of the 17,021 neonates admitted, 11,552 (67.9%) were inborn. During the three years reviewed, 1,814 (10.7%) neonates died. The mortality rates were 5.9% for inborn neonates, 24.1% for outborn neonates, and 4.8% for readmitted neonates. Mortality trends declined quarterly at an average of 0.15%. Factors associated with neonatal mortality included; birth weight < 2500g [{aRR = 1.14; 95% CI (1.01–1.29)}], GA < 37 weeks[{aRR = 1.17; 95% CI (1.03–1.33)}], fifth-minute Apgar score <7[{aRR = 2.53; 95% CI (2.32–2.77)}], vaginal delivery[{aRR = 1.39; 95% CI (1.24–1.55)}], out-born[{aRR = 2.85; 95% CI (2.58–3.14)}], positive maternal HIV status[{aRR = 1.18; 95% CI (1.02–1.36)}] and maternal age ≥35years [{aRR = 1.12; 95% CI (1.01–1.25)}].

**Conclusion:**

Despite a declining trend, in-hospital neonatal mortality at MNH is still high. Ongoing quality improvement strategies should prioritize the identification of neonates with risk factors for poor outcomes. Specifically, the high proportion of mortality among out-born neonates requires more attention and further investigation.

## Introduction

Globally, 2.4 million neonates died within their first month of life in 2019. Three-quarters of these deaths occur in the first week and more than one million in the first 24 hours. Neonatal deaths contribute approximately 47% to under-five mortality worldwide [1].

During the past two decades, the global neonatal mortality rate has decreased much slower, from 30 per 1000 live births in 2000 to 17 per 1000 live births in 2019. Most neonatal deaths occur in low-and-middle-income countries, with SSA and Asia contributing more than two-thirds to global neonatal mortality [2, 3]. In Tanzania, neonatal mortality remains high compared to other countries in SSA. The Tanzanian Government has implemented several programs and initiatives to reduce neonatal mortality. These interventions include the introduction of integrated management of childhood illnesses during the first week of life, expanded program of immunizations, increasing human resource in various health facilities and trainings on neonatal care such as essential newborn care and Basic Emergency Obstetric and Newborn Care (8). Furthermore, the Tanzanian government has prioritised the establishment of neonatal care units in all regional and district hospitals in the country.

These initiatives have resulted in an overall decline from 40 deaths per 1000 live births in 1999 to 20 deaths per 1000 live births in 2015–2016 [4].

The 2022 Tanzanian demographic health survey report indicates no substantial decline in the past seven years, with the current neonatal mortality reported at 24 per 1000 live births [5]. Neonatal mortality is still declining at a slower rate despite a significant increase in proportion of deliveries assisted by a skilled birth attendant from 66% in 2015–2016 to 85% in 2022, indicating the need to address other factors that contribute to neonatal mortality [5]. Several reports from referral hospitals in Tanzania show a high and stagnant in-hospital neonatal mortality [6] reflecting the need for improvement of quality care. With the current trend, Tanzania will not achieve the targeted 12 neonatal deaths per 1000 live births in 2030 [7].

The majority of neonatal deaths have been attributed to preterm birth complications, intrapartum-related complications (birth asphyxia or failure of spontaneous breathing at birth), infection, and birth defects [1]. Previous reports have identified birth weight, mode of delivery, place of delivery, gestation age (GA) and 5-minute Apgar score as potential risk factors for neonatal mortality [8, 9].

MNH neonatal unit is the largest in the country, and approximately 25% of neonates (in born and out born) of total monthly admissions die every month. Advances have been made in neonatal care over the past few years including expansion of the neonatal unit and having a fully functional neonatal intensive care unit. In addition, the number of health personnel has increased, and several staff members have participated in different trainings aimed at improving survival of neonates.

As we strive towards achieving the Sustainable Development Goal (SDG) indicator 3.2.2 [10], it is important to have adequate data at the facility level documenting trends and factors associated with neonatal deaths.

## Materials and methods

A hospital-based retrospective cohort study was conducted to determine trends and factors associated with neonatal mortality at MNH neonatal Unit in Dar-es-Salaam, Tanzania. MNH is a national referral hospital, research center and university teaching hospital that receives patients from different municipalities in Dar es Salaam. It also serves patients referred from other regions of the country. The neonatal unit is situated on the second floor of the maternity block and offers level III neonatal services with a total capacity of 130 cots. It is divided into neonatal intensive care unit (NICU), high dependency unit (HDU), and general wards (ward 36 and ward 37). Ward 36 is for term neonates and consists of subsections: room 1 (for care and sick neonates born at MNH), Room 2 (sick neonates referred from other facilities), room 3 (congenital anomalies) and isolation rooms. Ward 37 is for preterm neonates and consists of rooms 1 and room 2. The NICU and HDU have 7 and 15 cots, respectively. Admissions in the neonatal unit range from 15 to20 neonates per day with a monthly admission rate of 500 to 600 neonates. The unit is managed by 65 nurses, two neonatologists, nine pediatricians, five medical officers who have completed internship, rotating residents and intern doctors.

### Data collection

We reviewed records of all inborn and out-born neonates who were admitted at MNH from January 2018 to December 2020. All neonates less than 28 days who were admitted at the neonatal unit during the study period were included. Data were accessed from 1^st^ August 2021 to 20^th^ February 2022. The sources of data were the electronic medical record system and hard copy neonatal registers.

- Electronic medical record system (JEEVA system)
  MNH uses the JEEVA system (Napier Healthcare Solutions-India, version 4.3.2) for patient registration (outpatient and inpatient) and outpatient management including doctors’ consultation, prescriptions and medicine dispensing. However, for in-patient management, some of the documentation is still paper-based (e.g. patient notes) but the system is used for prescription, dispensing, requesting and receiving laboratory and radiological investigations, and generating discharge/death forms. All neonates admitted to the neonatal unit receive a unique hospital registration number that the JEEVA generates. The JEEVA system was used to determine the total number of neonates admitted from January 2018 to December 2020. If the neonate is re-admitted after discharge, the same hospital registration number is used; thus, all re-admissions are captured. The JEEVA system also reliably indicates the date of admission and date of discharge/death since all patients have to be discharged/removed from the system before leaving the hospital. Thus, the JEEVA system was used to extract the outcome i.e. alive or dead.
- Hard copy neonatal registers
  Additional variables that were not captured by the JEEVA system were extracted from the neonatal registers. Such information included maternal age and HIV status, mode and place of delivery, referring facility, date of admission, birth weight, sex, GA and Apgar score. Using the assigned unique registration number that was found both in the neonatal registers and the JEEVA system, the two data sets were then linked to obtain the final dataset that was used for analysis.

### Data management and analyses

Data was cleaned and analyzed using SPSS version 23. The dependent variable was neonatal death during the hospital stay. The independent variables were neonatal factors (birth weight, sex, Apgar score) and maternal factors (age, mode of delivery, place of delivery, HIV status, GA). Birth weight was divided into four categories based on WHO classification as follows: extremely low birth weight (ELBW), less than 1,000 grams (up to and including 999 grams); very low birth weight (VLBW), less than 1,500 grams (up to and including 1,499 grams); low birth weight (LBW), less than 2,500 grams (up to and including 2,499 grams); and normal birth weight, more than or equal to 2,500 grams (up to and including 3,999 grams). Neonates born with a birth weight of ≥4,000 grams are considered large for gestational age.

Continuous variables were summarized using median and interquartile range while categorical variables as numbers and or percentages. The Chi-square test was used to test for association between categorical variables.

The Poisson regression method was used to determine the relative risk with a 95% confidence interval. Any variable with a p value<0.05 at multivariate analysis was considered significantly associated with neonatal mortality. Multiple imputation was done on 10% of cases that had missing values. Multiple imputation is a computer method of estimating missing values in a data set. Multiple imputation by chained equation (MICE) was used. This was done with a linear model for numerical variables and logistic regression for categorical values. Only covariates (independent variables) were imputed.

The study obtained ethical clearance from Muhimbili University of Health and Allied Sciences (MUHAS) Institutional Review Board with approval number MUHAS-REC-05-2021-653. Then permission to conduct the study was obtained from the Directorate of Clinical Research, Training and Consultancy at MNH. The authors and research assistants’ accessed the original medical records and de-identified all patients’ information during extraction. The de-identified records were stored in password protected folders that could only be accessed by the authors and research assistants.

## Results

### Social demographic characteristics of study participants

**Fig 1:**
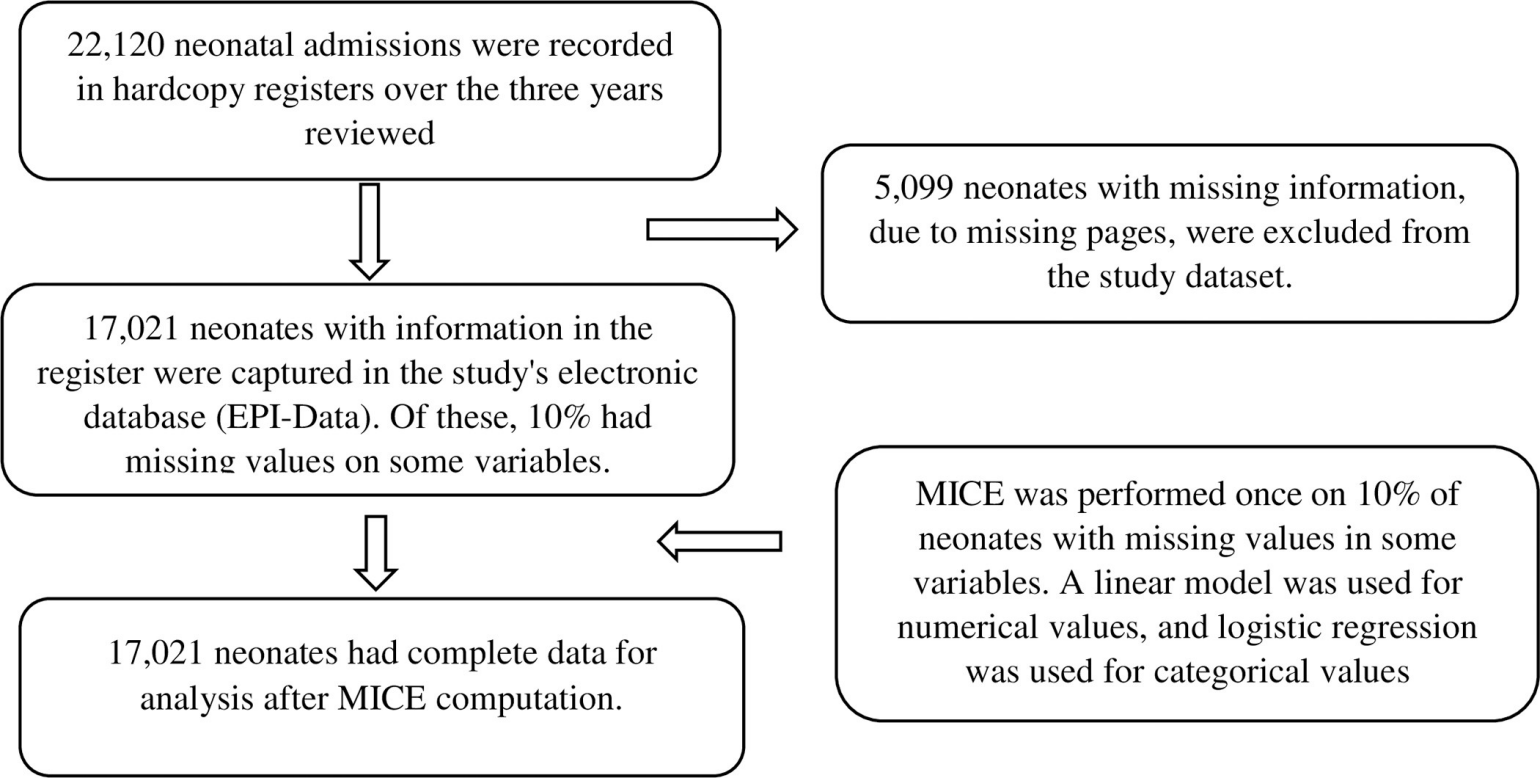
Flowchart showing inclusion of study participants.

Out of 22,120 neonatal admissions, only 17,021 were recovered and extracted from the neonatal registers (Fig 1). Of these, 11,552 (67.9%) were born at MNH (inborn). Most neonates were born to mothers aged 18–34 years, representing 80.8%. Caesarean section was the most common mode of delivery (56.5%), while 52.4% had a birth weight of ≥2,500 grams, 28.9% had low birth weight, and 3.6% had extremely low birth weight. Most neonates were born to mothers who were HIV-negative (93.8%), and more than half (56.9%) of the neonates were born prematurely (Table 1)

**Table 1:** Socio- demographic characteristics of the study participants (n = 17021).

			Neonatal status	
Variable		Frequency (%)	Survived n (%)	Died n (%)
**Age group (years)**				
	< 18	319 (1.9)	272 (85.3)	47 (14.7)
	18–34	13754(80.8)	12119 (88.1)	1635 (11.9)
	≥ 35	2948 (17.3)	2617 (88.8)	331 (11.2)
**HIV status**				
	Positive	833 (4.9)	688 (82.6)	145 (17.4)
	Negative	15968 (93.8)	14128 (88.5)	1840 (11.5)
	Unknown	220 (1.3)	192 (87.3)	28 (12.7)
**Gestational age in weeks**				
	< 28	598 (3.5)	353 (59)	245 (41)
	28–33	4402 (25.9)	3530 (80.2)	872 (19.8)
	34–36	4674 (27.5)	4254 (91)	420 (9)
	≥ 37	7347 (43.1)	6871 (93.5)	476 (6.5)
**Place of delivery**				
	MNH	11552 (67.9)	10873 (94.1)	679 (5.9)
	Referral	5284 (31)	4012 (75.9)	1272 (24.1)
	Home or born before arrival	185 (1.1)	123 (66.5)	62 (33.5)
**Mode of delivery**				
	Spontaneous vaginal delivery	7089 (41.6)	5697 (80.4)	1392 (19.6)
	Caesarean section delivery	9621 (56.5)	9063 (94.2)	558 (5.8)
	Assisted vaginal delivery	311 (1.9)	248 (79.7)	63 (20.3)
**Birth weight (gm)**				
	<1000	618 (3.6)	279(45.1)	339 (54.9)
	1000–1499	1641 (9.6)	1229 (74.9)	412 (25.1)
	1500–2499	4902 (28.9)	4474 (91.3)	428 (8.7)
	2500–3999	8926 (52.4)	8324 (93.3)	602 (6.7)
	≥4000	934 (5.5)	901 (96.5)	33(3.5)
**Apgar Score at 5 minutes**				
	<7	1456 (8.6)	893 (61.3)	563 (38.7)
	≥7	15565 (91.4)	14115 (90.7)	1450 (9.3)
**Sex**				
	Male	8971(52.7)	7855 (87.6)	1116 (12.4)
	Female	8029 (47.2)	7140 (88.9)	889 (11.1)
	Undetermined	21 (0.1)	13 (61.9)	8 (38.1)
**Readmissions**				
**Yes**		815 (4.8)	776 (95.2)	39 (4.8)
**No**		16206 (95.2)	14392 (88.8)	1814 (11.2)

Key: MNH:-Muhimbili national Hospital, HIV:-Human Immunodeficiency Virus

### Overall mortality among admitted neonates

**Fig 2:**
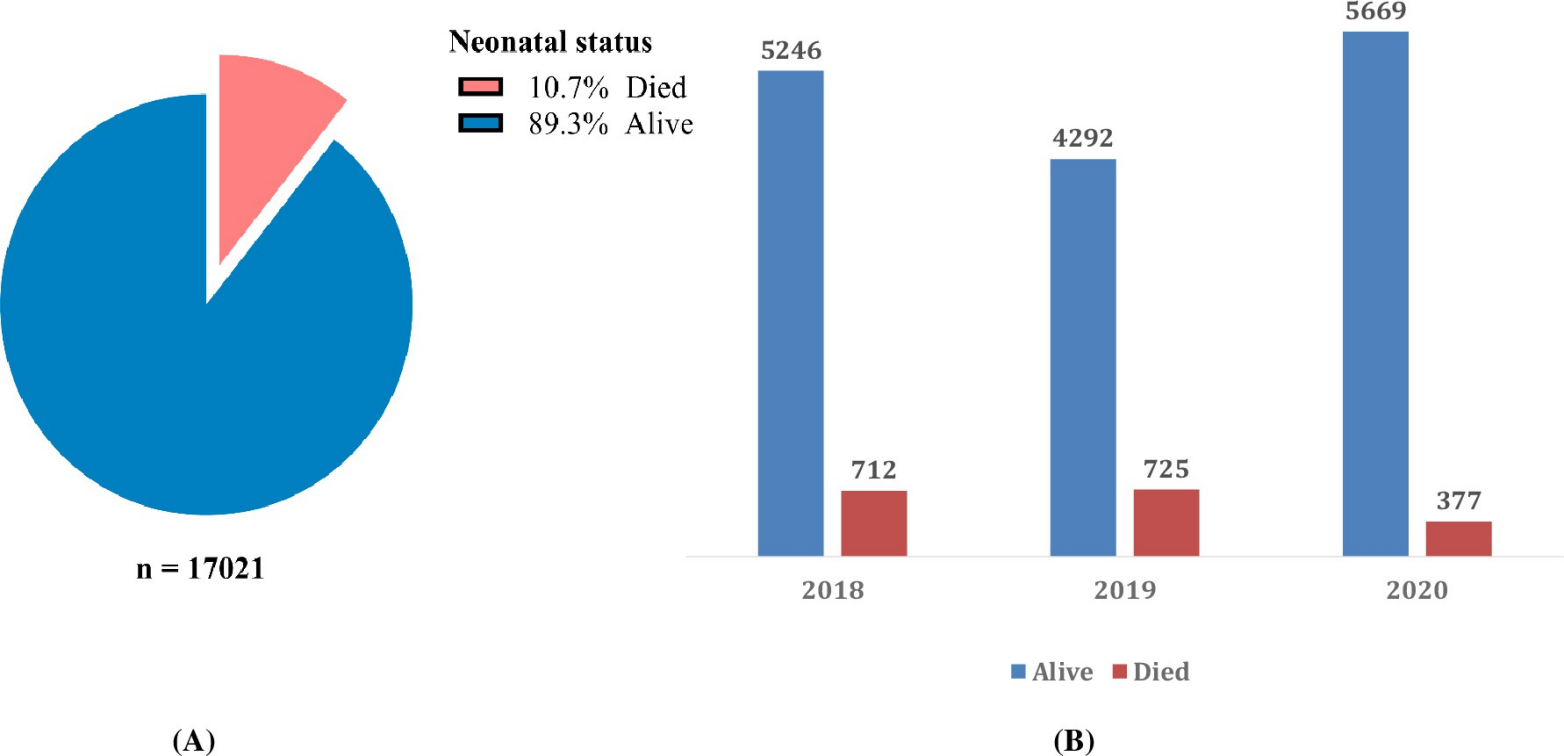
Overall and yearly distribution of mortality among admitted neonates.

During the three years reviewed, 1,814 neonates died, representing an overall mortality of 10.7% (Fig 2A). In addition, 39 readmitted neonates died, representing 0.2%. Analyzing the yearly neonatal mortality rates reveals noteworthy trends. In 2018, there were 712 neonatal deaths, constituting 12.0% of the total occurrences. The following year, 2019, saw an increase in neonatal mortality to 725 cases, accounting for 14.5% of the total. However, a notable decline occurred in 2020, with only 377 reported neonatal deaths, representing 6.2% of the total occurrences (Fig 2B).

### Trends of neonatal admissions and mortality

**Fig 3:**
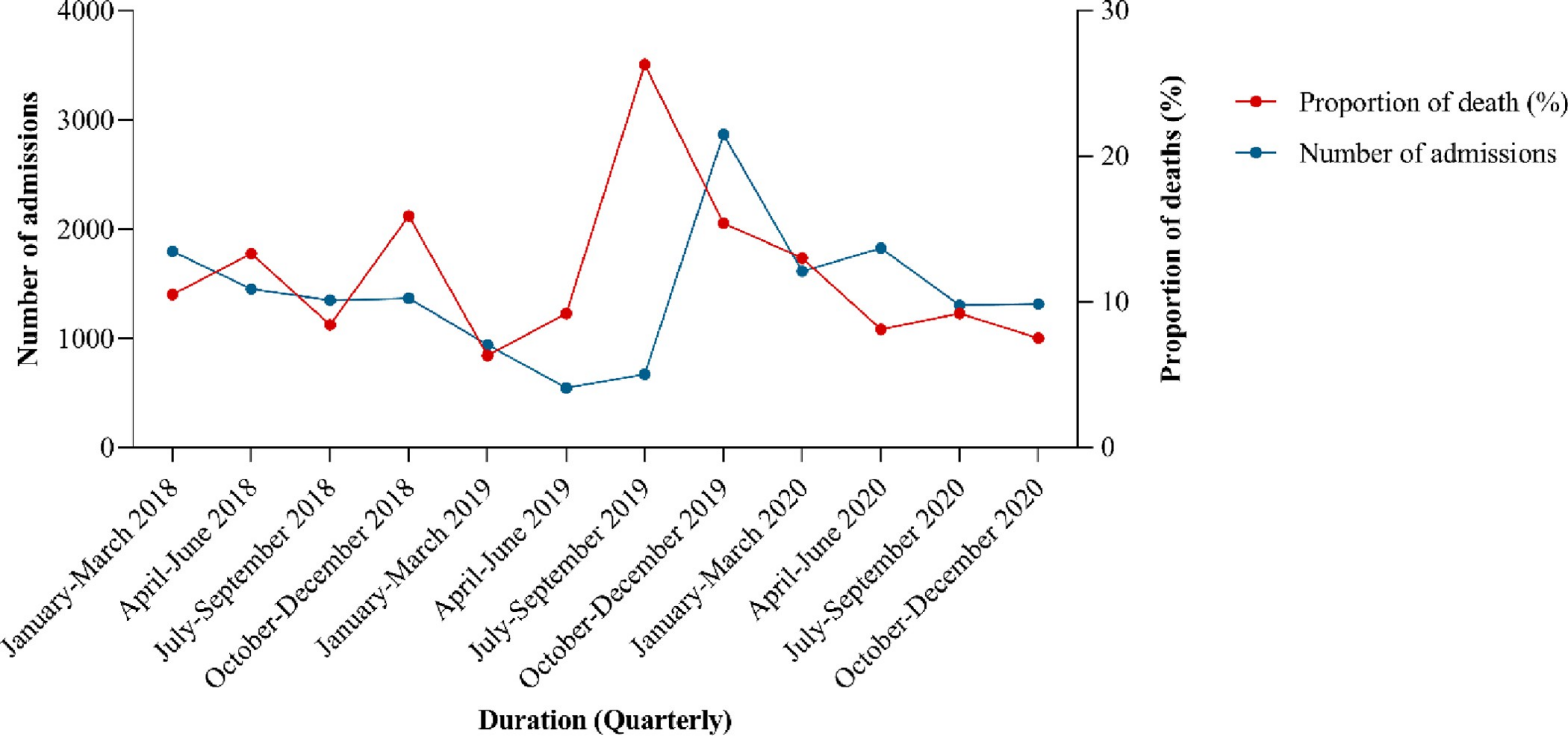
Trends of neonatal admissions and mortality.

Fig 3 shows the trend of admissions and mortality from January 2018 to December 2020. In the first quarter of 2018, admissions were initially high, just below 2,000, but declined to as low as under 1,000 by the second quarter of 2019. From the third quarter of 2019, admissions began to rise, peaking at 3,000 in the last quarter of 2019, before starting to decline again through the last quarter of 2020. Mortality trends exhibited more fluctuations, with neonatal mortality increasing on average from the first quarter of 2018, peaking in the third quarter of 2019, and then declining to below 10% in 2020.

**Fig 4:**
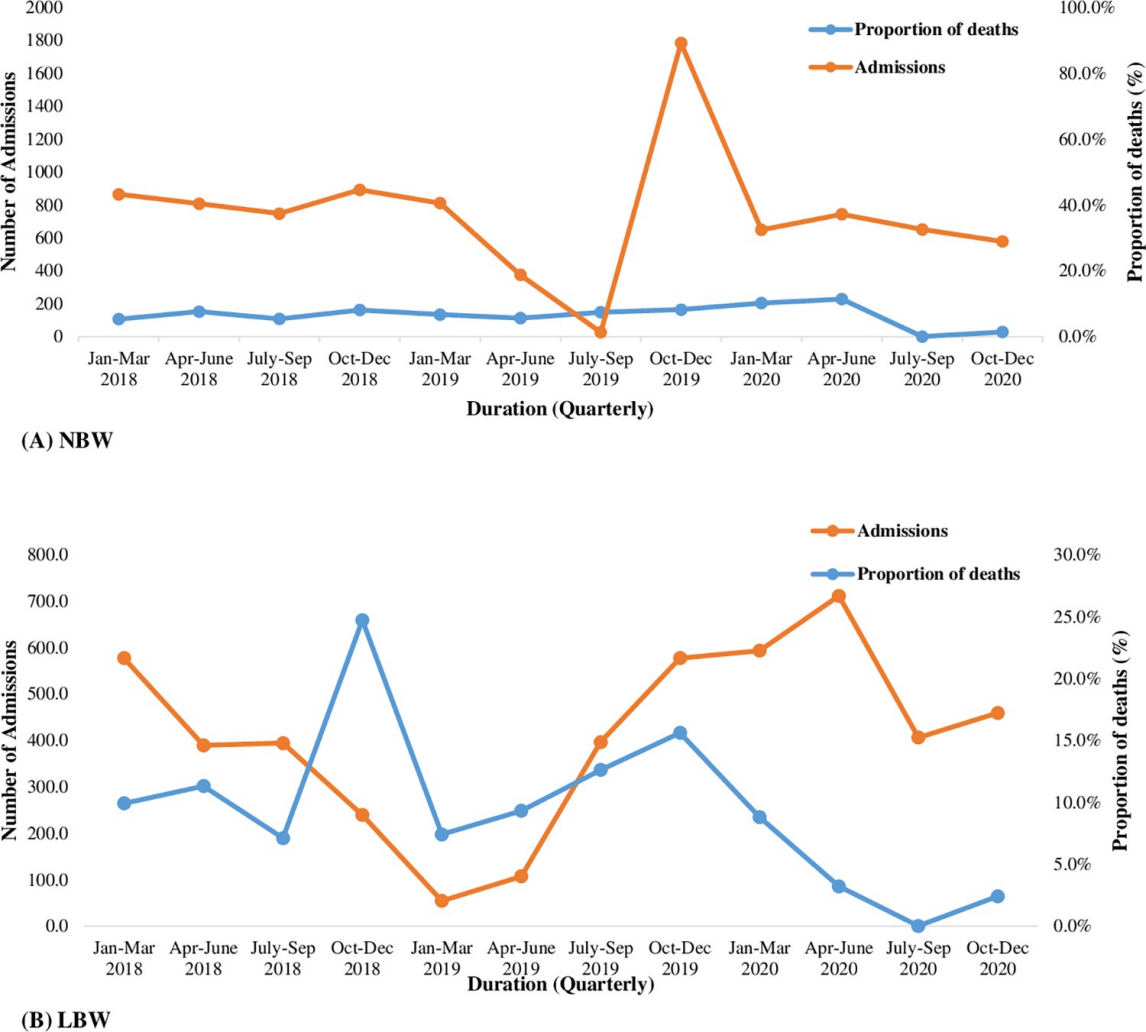
Trends of neonatal admissions and mortality based on birth weight.

Fig 4 shows the trend of admissions and mortality based on birth weight. Most admissions were of normal birth weight (NBW, ≥2,500–3,999 g), except in the third quarter of 2019, when admissions of low birth weight (LBW, <2,500 g) exceeded those of NBW. The proportion of deaths among LBW neonates has consistently been higher than that among NBW neonates, except in the last quarter of 2019, when a reduction was observed and remained constant.

**Fig 5:**
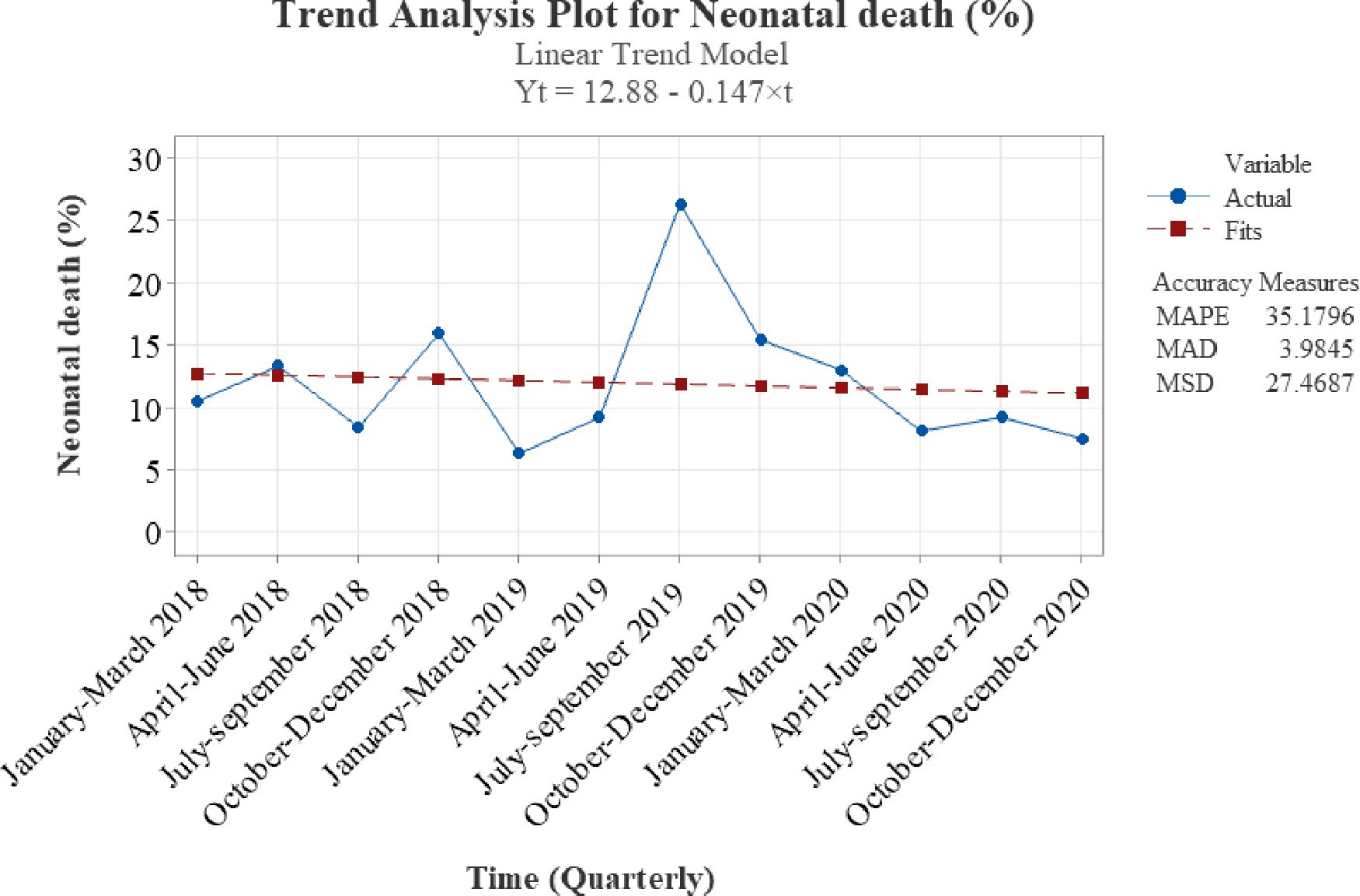
Trend and average quarterly decline in neonatal mortality.

Fig 5 shows the trend of mortality, with the dotted lines in the graph representing the average decline in neonatal mortality of 0.15% each quarter.

### Factors associated with neonatal mortality

The Poisson regression model was used to determine the relative risk of mortality with corresponding 95% confidence intervals (CIs). Neonates born at a GA of less than 37 weeks were more likely to die compared to those born at a GA of 37 weeks or greater. Similarly, out-born neonates were more likely to die compared to inborn neonates (adjusted relative risk [aRR] = 2.85; 95% CI [2.58–3.14], p < 0.001). Extremely low birth weight (ELBW) neonates had a threefold increased risk of dying compared to neonates of normal birth weight (NBW) (aRR = 3.20; 95% CI [2.74–3.73], p < 0.001). On the other hand, neonates with a birth weight greater than 4000 g were 0.97 times less likely to die compared to NBW neonates. Neonates with a fifth-minute Apgar score of less than 7 were 2.5 times more likely to die compared to those with a fifth-minute Apgar score of 7 or greater (aRR = 2.53; 95% CI [2.32–2.77], p < 0.001) (Table 2).

**Table 2:** Univariate and multivariate analyses of factors associated with neonatal mortality.

		Univariate analysis			Multivariate analysis		
Variable		cRR	95% CI	*P*-value	aRR	95% CI	*P*-value
**Maternal factors**							
**Maternal age (years)**							
	< 18	1.24	0.95–1.62	0.116	1.03	0.81–1.30	0.822
	≥ 35	0.95	0.85–1.06	0.315	1.12	1.01–1.25	0.029
	18–34	Ref					
**Maternal HIV status**							
	Positive	1.06	1.03–1.09	< 0.001	1.18	1.02–1.36	0.029
	Unknown	1.01	0.97–1.06	0.594	0.77	0.54–1.10	0.151
	Negative	Ref					
**GA (weeks)**							
	< 28	6.32	5.56–7.20	< 0.001	1.26	1.05–1.51	0.011
	28–33	3.06	2.75–3.40	< 0.001	1.43	1.25–1.64	< 0.001
	34–36	1.39	1.22–1.57	< 0.001	1.17	1.03–1.33	0.014
	≥ 37	Ref					
**Place of delivery**							
	Referral	4.10	3.75–4.47	< 0.001	2.85	2.58–3.14	< 0.001
	Home or BBA	5.70	4.60–7.07	< 0.001	2.91	2.34–3.62	< 0.001
	MNH	Ref					
**Mode of delivery**							
	Spontaneous Vaginal Delivery	3.39	3.08–3.72	< 0.001	1.39	1.24–1.55	< 0.001
	Assisted vaginal delivery	3.49	2.76–4.42	< 0.001	1.53	1.20–1.95	0.001
	C/S	Ref					
**Neonatal factors**							
**Birth weight (grams)**							
	< 1000	8.02	7.28–8.83	< 0.001	3.20	2.74–3.73	< 0.001
	1000–1499	3.85	3.47–4.28	< 0.001	2.24	1.95–2.57	< 0.001
	1500–2499	1.33	1.19–1.49	< 0.001	1.14	1.01–1.29	0.030
	2500–3999	Ref					
	≥ 4000	0.98	0.90–1.30	0.365	0.97	0.92–1.06	0.748
**APGAR score at 5 minutes**							
	< 7	4.15	3.83–4.50	< 0.001	2.53	2.32–2.77	< 0.001
	≥ 7	Ref					

Key: cRR: crude Relative Risk, aRR: adjusted Relative Risk, Ref: Reference category; GA: Gestational Age

## Discussion

This report describes trends in neonatal mortality and associated factors over a three-year period from the largest tertiary neonatal unit in Tanzania. The overall mortality rate was found to be 10.7%. There were wide variations in admissions and mortality over time. The trend in annual mortality rates shows an increase from 12% in 2018 to 14% in 2019, followed by a notable decline to 6.2% in 2020. Overall, a declining trend in mortality, averaging 0.15% quarterly, was observed. Prematurity, low birth weight, a low fifth-minute Apgar score, being out-born, vaginal delivery, positive maternal HIV status, and advanced maternal age were associated with higher neonatal mortality.

The mortality revealed in this study was lower than the 20.2% recorded at a teaching hospital in Ghana [8], and slightly higher than the 10% recorded in Cameroon [11]. However, at our facility, mortality among referred neonates remains high and contributes a higher proportion to overall mortality. Out-born neonates are usually very sick, and mainly transported without proper stabilization before and during the transfer resulting in unstable admissions. Other contributing factors include lack of essential monitoring tools during transportation and being transported in ambulances that are not fully equipped as observed by Kiputa *et al* [12]. Additionally, 4.8% of readmitted neonates died likely reflecting early discharge in a mostly overcrowded facility. Previous reports elsewhere indicate jaundice, sepsis, poor feeding, early hospital discharge and respiratory problems as common reasons for readmissions [13, 14]. There is need to invest more in health systems, including infrastructure, human resources and equipment coupled with active use of local data to steer both coverage and quality.

In this report, the trend of neonatal mortality has shown a wide variation from 2018 to 2020 including a sharp rise in 2019 which coincides with an increase in neonatal admissions. The infrastructure and renovations for establishing the neonatal intensive care unit at MNH were completed in 2019 however it became fully operational with equipment in 2020. It was further noted that there was a surge in admissions of premature neonates during the first three quarters of 2019. The opening of the NICU likely contributed to the rise in admissions from referring facilities with the hope of increasing the chance of survival of LBW neonates. This is consistent with the surge in mortality during the same time as prematurity and LBW are both risk factors of neonatal mortality.

The Covid 19 pandemic also significantly disrupted neonatal care services in Dar es Salaam. One of the district hospitals was converted to a Covid 19 specialized centre, and its neonatal unit was closed. High volume health centres were allowed to refer patients directly to MNH, which is reflected in a rise in admissions in 2019 (Fig 3). However, the overall mortality seemed not to be affected possibly due to more stable neonates being referred in addition to those sicker that would have been referred before the pandemic anyway.

Overall, the trend in mortality has shown a steady decline at an average of 0.15% quarterly since 2018 which could be explained by the continued local efforts invested in improving neonatal care. The use of continuous positive airway pressure (CPAP) and operationalization of the neonatal intensive care unit went hand in hand with training of the healthcare providers and an increased number of providers allocated to the neonatal intensive care unit. There was also a randomized controlled trial on the impact of starting kangaroo mother care (KMC) immediately after birth compared to routine KMC after stabilization of the neonate. The study trained all health care providers in the unit on the WHO minimum package of care for LBW neonates. The study showed an increase in survival in the intervention group, which likely contributed to a further decrease in neonatal mortality [15]. Other low-and middle-income countries have reported a steady drop in neonatal mortality in their respective facilities after improvement of working conditions [11, 16].

The mortality of premature neonates i.e. GA < 37 completed weeks was significantly higher than their counterparts born at term. An inverse relationship was observed between mortality and gestational age. Similar findings were observed in studies conducted in other low- and middle-income countries [17–20]. Prematurity predisposes to several complications such as respiratory distress syndrome (lack of surfactant), apnea of prematurity (immaturity of the central respiratory drive and breathing muscles), pulmonary hemorrhage, feeding intolerance and hypoglycemia which contribute to increased risk of neonatal mortality [21]. Our study also showed that LBW was associated with increased risk of mortality as compared to NBW. The highest mortality was seen in smaller neonates weighing <1000g. Fort *et al* and Reyesa *et al* reported similar findings [22, 23]. LBW neonates have immature organs and systems which makes them susceptible to infections, hypothermia, respiratory distress syndrome, apnea, hypoglycemia and intra-ventricular hemorrhage.

It is well known that vaginal delivery is safe for both fetus and mother at term. In this report, vaginal delivery was associated with an increased risk of neonatal mortality. It was also noted that many LBW neonates were born via vaginal delivery. Pedro *et al* reported that LBW neonates had an increased risk of vaginal breech delivery, and a reduced possibility of caesarian section due to cephalo-pelvic disproportion [24]. LBW itself is an independent predictor of mortality and likely, responsible for the association of vaginal delivery and mortality. Mbawala *et al*, reported similar findings in similar settings [25]. Vaginal delivery prepares the fetus for extra uterine life through physiological changes in homeostasis, respiratory and cardiovascular system. However, vaginal delivery has also been shown to be associated with poor outcome for premature neonates. Therefore, this association between vaginal delivery and mortality should be interpreted with caution.

Neonates that are born with an Apgar score of <7 at five minutes usually have a higher risk of death compared to those that have a normal Apgar score. These findings were observed in our study as well as similar studies conducted in Cameroon, Brazil and Eritrea [17, 26, 27]. Neonates with severe birth asphyxia have multiple organ dysfunction including convulsions, respiratory distress and gastrointestinal hemorrhage which ultimately lead to their death. Although Apgar score is known to be subjective and sometimes unreliable, in most low- and middle-income countries it can be the only proxy for asphyxia apart from delayed onset of spontaneous breathing [28].

We report a positive maternal HIV status to be associated with increased risk of neonatal mortality. Similar findings from studies done in Ethiopia and Rwanda also showed a higher mortality among neonates born to mothers living with HIV [29–31]. HIV infection is known to increase the risk of prematurity which is one of the major causes of neonatal mortality. In addition, exposed neonates are predisposed to HIV and opportunistic infections that contribute to their death.

Maternal age ≥35 years was also associated with an increased risk of neonatal mortality. Other studies have also found an association between maternal age and neonatal mortality [30, 32]. Advanced maternal age is associated with increased risk of congenital malformations and chronic diseases such as hypertension and diabetes that predispose to premature birth which increases the risk of neonatal death [33].

### Strength of the study

The study’s large sample size and inclusion of all neonates admitted to the MNH neonatal unit during the three years reviewed increased the precision of results, reduced chances of selection bias and increased applicability of the findings to other referral health facilities in Tanzania. The study established mortality for in-born and out-born neonates and their associated factors, which has not been done in previous studies conducted at our facility.

### Study limitations

Exposure variables studied were limited to routinely collected factors and there was missing data due to incompleteness of the registers. Neonates completely missing from the registers (n = 5099) were not analysed which could have led to an underestimation of overall mortality.

## Conclusion

Overall facility mortality was 10.7% and the trend in neonatal mortality declined at a rate of 0.15% quarterly. Birth weight <2500g, GA <37 weeks, fifth-minute Apgar score <7, vaginal delivery, positive maternal HIV status, out-born and maternal age ≥ 35 years were all associated with increased risk of neonatal mortality.

### Recommendation

Implementation of multifaceted interventions should prioritize neonates with the identified risk factors in order to further reduce neonatal mortality.

## Supporting information

S1 Checklist (DOCX)

S1 Dataset (XLSX)
