# Supplementary material for: Trends in mortality and associated factors among neonates hospitalized at Muhimbili national hospital, Tanzania: A three-year retrospective study
Source: PLoS One. 2024 Nov 8;19(11):e0310256. doi: 10.1371/journal.pone.0310256 (PMC11548724; doi:10.1371/journal.pone.0310256)
Supplement: S1 Checklist — (DOCX) [file pone.0310256.s001.docx]

**Appendix I: checklist**

Study ID……………

Date of birth..…/…../…….

Date of admission……/…../…..

Date of death…../…../…….

**A.MATERNAL FACTORS**

1. Age
2. Place of delivery
3. MNH
4. Referred
5. Name of facility…………………………..

3) Mode of delivery

a) SVD

b) Assisted vaginal delivery

c)Caesarean section

4) Gestation at delivery……………….weeks

5) HIV status

a)positive

b)negative

c)unknown

**B.NEONATAL FACTORS**

6) Birth weight ………gms

7) Gender

a) Male

b) Female

8) Apgar score

a) 1^st^ minute……

b) 5^th^ minute…..
